# Endobronchial deposits of chronic lymphocytic leukemia – an unusual cause of central airway obstruction

**DOI:** 10.1002/rcr2.96

**Published:** 2015-02-25

**Authors:** Miranda Maw, Michael Harvey, Zinta Harrington, Melissa Baraket, Renn Montgomery, Jonathan Williamson

**Affiliations:** 1 Department of Respiratory Medicine, Liverpool HospitalSydney, New South Wales, Australia; 2 Department of Haematology, Liverpool HospitalSydney, New South Wales, Australia; 3 Department of Anatomical Pathology, Liverpool HospitalSydney, New South Wales, Australia

**Keywords:** Bronchoscopy, central airway obstruction, chronic lymphocytic leukemia, endobronchial deposits

## Abstract

A 66-year-old woman with a background of chronic lymphocytic leukemia (CLL) was admitted to the hospital on several occasions with recurrent episodes of community-acquired pneumonia. Computed tomography and bronchoscopy revealed multiple obstructing endobronchial polyps. Post-obstructive pneumonia together with immunoglobulin G deficiency was considered the most likely cause of these recurrent infections. Bronchoscopy was performed for removal of the critically obstructing lesions. Histopathology revealed replacement of bronchial mucosa with CLL deposits. Despite a brief window of infection-free survival following therapy, she remained susceptible to pneumonia with further hospital admissions and eventually died from her disease.

## Introduction

Respiratory complications are common in patients with chronic lymphocytic leukemia (CLL) and can result in significant morbidity and mortality [1]. Although pulmonary involvement can be seen in up to 40% of autopsy cases in patients with CLL, only a small percentage manifest clinical or radiologic evidence of pulmonary involvement in life [2]. Pneumonia is the most common pulmonary complication followed by malignant pleural effusion and upper respiratory tract infection [1]. Other pulmonary complications include extrinsic central airway obstruction from hilar and mediastinal lymphadenopathy, pulmonary leukostasis and leukemic infiltrates [1]. To our knowledge, endobronchial polyps of CLL tumor have not previously been reported.

## Case Report

**Figure 1:**
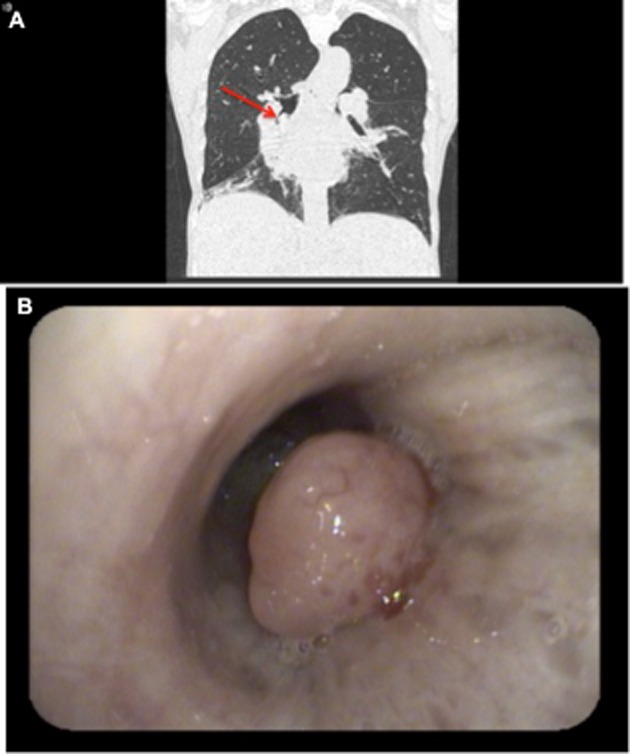
(A) Computed tomography image showing multiple nodular lesions causing varying degrees of obstruction in the central airways. Note the obstructing polyp in the bronchus intermedius (arrow). (B) A chronic lymphocytic leukemia nodular lesion causing obstruction of the bronchus intermedius lumen.

A 66-year-old woman of Middle-Eastern background presented with fevers, productive cough, and coryzal symptoms to a tertiary referral center and was diagnosed with right lower lobe community-acquired pneumonia. She had had three similar presentations within the previous year, leading to lengthy hospital admissions for treatment. A computed tomography chest (Fig. 1A) showed right lower lobe and lingula pneumonia along with multiple nodular lesions within the central airways causing varying degrees of obstruction.

Her past medical history included CLL diagnosed 7 years prior and managed on low-dose prednisone and regular intravenous immunoglobulin transfusions for associated immunoglobulin G deficiency, chronic hepatitis B, chronic obstructive pulmonary disease, active smoking, and recurrent urinary tract infections.

The CLL had not responded to chlorambucil and rituximab nor had she shown a clinical response to steroid therapy despite a prolonged trial. Because of her poor performance status (Eastern Cooperative Oncology Group performance status of 2), there was concern about her tolerance of further immunosuppressive chemotherapy particularly given her poor response thus far. She was therefore referred for therapeutic endobronchial resection of the tumor deposits to reduce the risk of recurrent infection and airway collapse.

At bronchoscopy, multiple endobronchial polyps were present. A lesion on the postero-lateral wall of the proximal left main bronchus was causing noncritical obstruction. A second lesion, also non-obstructive, was noted in the left upper lobe. On the right, a polyp was present in the apical segment of the right upper lobe. A larger lesion was present in the distal bronchus intermedius (BI) along the posterior wall causing 60–70% obstruction of the right lower lobe lumen (Fig. 1B). This was resected using a snare diathermy and another lesion, arising from the RB6 segment, was noted and similarly resected. Six freeze–thaw cycles of cryotherapy were then applied to the base of the BI lesion. The patient tolerated the procedure well and was discharged home 2 days post bronchoscopy.

**Figure 2:**
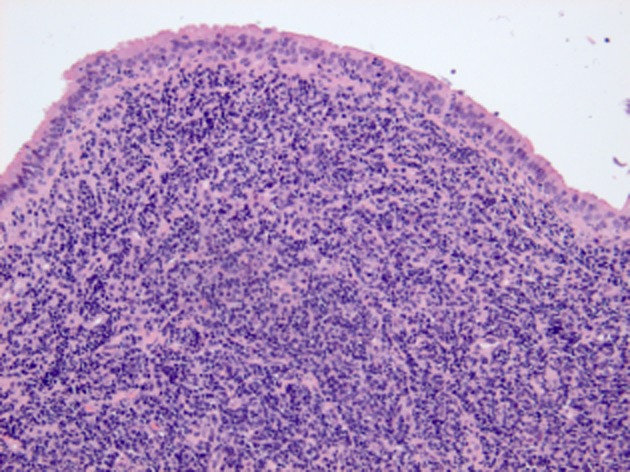
Haematoxylin and eosin stain demonstrating the snared bronchus intermedius polyp consisting of a large deposit of chronic lymphocytic leukemia with overlying respiratory epithelium.

Histopathology revealed diffuse infiltration of small lymphocytes in the polyps consistent with CLL involvement (Fig. 2).

The patient was free of cough and infection for 2 months, but then developed two episodes of post-obstructive lingula pneumonia. She underwent a second bronchoscopy 4 months after the first and a polyp causing complete occlusion of the lingula was snared and debulked. The tumour in the right BI had regrown now causing 50% narrowing of the airway – this was treated with freezing cycles of cryotherapy. Given her declining health, no further endobronchial interventions were undertaken. The patient was discharged on home oxygen under the care of the palliative team for end of life care and died 2 months after the second bronchoscopy.

## Discussion

CLL accounts for approximately 30% of all leukemias and is the commonest form of leukemia among older populations in developed countries [3]. CLL is a malignancy of mature B cells with typically the co-expression of the CD5, CD20, and CD23 antigens [4]. Although the etiology of CLL is unknown, recognized risk factors include male sex, advanced age, white race, and a family history of CLL [3]. Available treatments generally induce remission, although nearly all patients relapse, and CLL remains an incurable disease [4].

Combination chemoimmunotherapy with fludarabine, cyclophosphamide, and rituximab is considered first line in the treatment of CLL, with overall response rates of 95%, and complete remission rates of 44% [5]. However, this treatment is too toxic for many elderly patients, who constitute most of the individuals with this disease, and there remain subgroups of patients for whom this therapy has minimal effect [5].

CLL can have several pulmonary manifestations that are often difficult to distinguish from other pulmonary disorders on clinical grounds alone [2]. Common radiographic findings include pulmonary infiltrates, pleural effusion, and hilar and mediastinal lymphadenopathy [2]. The most common cause of infiltrates seen in patients with CLL was found to be secondary to infections [2].

Berkman et al. described three cases of pulmonary infiltration with CLL with clinical and radiological evidence of pulmonary disease due to leukaemic infiltration [2]. The diagnosis of pulmonary CLL in all three patients was established through transbronchial biopsy, and, in two cases, bronchoalveolar lavage [2]. These patients were commenced on chemotherapy and steroid therapy with varying responses to the antileukaemic treatment [2]. A single case report by Chernoff et al. in 1984 detailed the course of a patient with endobronchial CLL causing narrowing and edema of the left upper and lower lobe bronchi resulting in recurrent post-obstructive pneumonia [6]. However, the narrowing was diffuse and not associated with focal polypoid deposits of CLL as in our case.

Airway obstruction does not always require intervention, but in a well-selected population airway interventions could be considered to offer therapeutic benefit when other modalities of treatment are limited [6]. Interventional procedures can offer immediate symptom relief and improved quality of life with minimal risks and peri-procedural complications [7]. Several techniques are available to manage central airway obstruction and include airway dilatation, ablation, and tracheobronchial stenting [7]. The risk–benefit profile of such interventions need to be weighed carefully as in our patient for whom a palliative intent procedure was deemed important to improve quality of life, albeit briefly.

## Disclosure Statements

No conflict of interest declared.

Appropriate written informed consent was obtained for publication of this case report and accompanying images.
